# Study on acupuncture improving insomnia comorbid with depression and anxiety based on rs-fMRI: A protocol for systematic review and meta-analysis

**DOI:** 10.1097/MD.0000000000025988

**Published:** 2021-05-21

**Authors:** Lin Yao, Mengyuan Li, Jiazhen Cao, Yanze Liu, Haizhu Zheng, Haipeng Huang, Hongfeng Wang

**Affiliations:** Changchun University of Chinese Medicine, Nanguan District, Changchun, Jilin, China.

**Keywords:** acupuncture, anxiety, depression, insomnia, meta-analysis, protocol, rs-fMRI, systematic review

## Abstract

**Background:**

Long term insomnia and low sleep quality often lead to depression, anxiety and other negative emotions, and often interact with each other. Many studies have confirmed the effectiveness of acupuncture in the treatment of insomnia comorbid with emotional disorders, but its specific mechanism needs to be further explored. Resting-state functional magnetic resonance (rsfMRI) is an important means to study the changes of brain activity. However, the results are inconsistent and lack of systematic evaluation and analysis.

**Methods:**

Nine databases will be searched, including PubMed, EMBASE, EBSCOhost-medline, Web of Science, Cochrane Library, China National Knowledge Infrastructure, VIP Database and Wan-Fang Database, Chinese Biomedical Literature Database from inception to January 2021. And screening clinical registration platform related research, in order to obtain more relevant studies. The outcomes include the change of rs-fMRI, sleep quality, depression, and anxiety. Quality assessment of the included studies will be performed according to the Cochrane Risk of Bias tool. Evidence quality will be assessed by using the Grading of Recommendations Assessment, Development, and Evaluation (GRADE) method. RevMan software (Version 5.3) and stata13.1will be used for statistical analyses. Subgroup analysis will be performed if necessary. If the data is insufficient, qualitative synthesis will be conducted instead of quantitative synthesis.

**Results:**

This study will analyze the effect of acupuncture on the brain activity changes, improvement of sleep quality and clinical symptoms of anxiety and depression with insomnia comorbid with emotional disorders.

**Conclusion:**

This study used meta-analysis method to explore the characteristics of acupuncture on brain activity changes in insomnia comorbid with emotional disorders, so as to provide effective evidence for clarifying its pathogenesis.

## 1 Introduction

Sleep is a universal function of living species and represents one of the most important psychophysiological processes for brain function and mental health.^[1]^ Sleep time occupies up to a third of the human lifespan.^[2]^ With the increasing pressure of external factors such as modern society, environment, and working mode, the incidence rate of insomnia disorder is increasing year by year. Insomnia is a subjective feeling that sleep time and sleep quality do not meet daytime social function, accompanied by symptoms such as irritability or fatigue during wakefulness.^[3]^ Prevalence estimates for insomnia symptoms range between 30% and 50%^[4]^ in the general adult population and up to 80% in patients with psychiatric illnesses.^[5]^ There is a complex two-way relationship between insomnia and negative emotions such as depression and anxiety, which interact with each other, are relatively independent.^[6]^ Long term insomnia or low sleep quality will lead to the imbalance of the interaction between sympathetic and parasympathetic nerves, and cause anxiety, depression, and other negative emotions.^[7]^ When the body is in a state of tension and anxiety for a long time, the sensory function of the nervous system will be extremely sensitive, causing difficulty in falling asleep, easy to wake up, aggravating sleep disorders, forming a vicious circle.^[8,9]^ Research using resting-state functional magnetic resonance imaging (rs-fMRI) suggests that insomnia is related to arousal, emotion, reward, and abnormal functional activities in cognitive brain regions.^[10,11]^ It is not only related to functional abnormalities in local brain regions, but also related to abnormal internal connections of default network, prominence network, and emotional network.^[12]^

At present, novel coronavirus (COVID-19) pneumonia is in the world. Due to less doctors and more patients, high work intensity and other reasons, the proportion of sleep in medical workers is seriously imbalanced, which is more likely to lead to insomnia and other sleep problems.^[13]^ According to the latest sleep survey report released by the China Sleep Research Association, at present, more than 300 million people in China have sleep disorders, and the COVID-19 epidemic has delayed falling asleep for 2 to 3 hours. Long term insomnia can affect the emotion, learning, memory, and other functions of medical workers, resulting in the decline of work efficiency and the increase of error rate.^[14]^ Therefore, how to reduce the negative impact of insomnia and other sleep problems on medical workers and improve the quality of medical service has become the key problem to be solved urgently.

Acupuncture is one of the important complementary and alternative therapies. Many studies have confirmed the effectiveness of acupuncture in the treatment of insomnia comorbid with depression and anxiety,^[15–17]^ but its specific mechanism needs to be further explored. Rs-fMRI is an important means to study the neural mechanism, because of its good repeatability, noninvasive, and other advantages, it has become the preferred method of modern neuroimaging research. In rs-fMRI technology, by using the analysis method of amplitude of low-frequency fluctuations (ALFF) and regional homogeneity (ReHo), it can reflect the changes of spontaneous brain activity more stably and accurately in the resting state. Some studies have explored the characteristics and rules of brain activity changes in insomnia comorbid with depression and anxiety patients treated with acupuncture by calculating the changes of ALFF and Reho in the brain. However, the results are inconsistent due to the differences of inspection equipment, test standards of analysis software and sample size. Therefore, this study used meta-analysis method to integrate and analyze the changes of ALFF and Reho, and to explore the characteristics and rules of acupuncture on the changes of brain activity in insomnia comorbid with emotional disorders, in order to provide effective evidence for the elucidation of its pathogenesis.

## 2 Methods

The protocol of this meta-analysis will be conducted and reported in accordance with the Preferred Reporting Items for Systematic Reviews and Meta-Analysis Protocols statement guidelines.^[18]^ The protocol has been registered on International Platform of Registered Systematic Review and Meta-analysis Protocols, and the trial registration number is CDR42021235039.

### 2.1 Criteria for including studies in the review

#### 2.1.1 Types of studies

The review will include randomized controlled trials reporting that study on acupuncture improving depression and anxiety in patients with insomnia based on rs-fMRI. Randomized controlled trials comparing pharmacotherapy, sham-acupuncture, or placebo will be included. All eligible trials will be included regardless of language and publication types. Articles of the following research types will be excluded: case series, observational studies (including cohort and case-control studies), and retrospective studies, qualitative studies, animal experiments, review articles. There are no restrictions on study area, race, patient age, and gender.

#### 2.1.2 Types of participants

Participants who meet domestic and internationally recognized diagnostic criteria for insomnia will be included, like International Classification of Sleep Disorders Third Edition^[19]^ or Chinese Classification of Mental Disorders Version 3.^[20]^ Regardless of age, gender, and source of cases. And patients need to be accompanied by a certain degree of emotional disorder pathological anxiety and depression (secondary to insomnia).

#### 2.1.3 Types of interventions

The intervention methods include body acupuncture (manual/electro), auricular acupuncture, and scalp acupuncture, regardless of needling techniques and stimulation method. The intervention without stimulating acupoints and combined with other treatments were excluded.

#### 2.1.4 Types of outcomes

The outcomes include whole-brain regional homogeneity (ReHo), amplitude of low-frequency fluctuations (ALFF), and evaluated using fMRI; changes of sleep quality, assessed using Pittsburgh Sleep Quality Index and Insomnia Severity Index ; changes of depression and anxiety, assessed using Hamilton Depression Scale, Hamilton Anxiety Scale.

### 2.2 Search methods for identification of studies

#### 2.2.1 Electronic searches

The following 9 databases will be searched from inception to January 2021, namely PubMed, EMBASE, EBSCOhost-medline, Web of Science, Cochrane Library, China National Knowledge Infrastructure, VIP Database and Wan-Fang Database, Chinese Biomedical Literature Database. The search term will consist of 4 parts: study type, disease, intervention method, and technical means: (“randomized controlled trial” or “randomised” or “randomly” or “trial” or “clinical trials”) and (“insomnia” or “dyssomnias” or “sleep disorders” or “sleep loss” or “sleep insufficient” or “sleep deprivation”) and (“depression” or “anxiety” or “emotional disorders”) and (“acupuncture therapy” or “acupuncture” or “acupoints” or “body acupuncture” or “scalp acupuncture” or “manual acupuncture” or “auricular acupuncture” or “ear acupuncture”) and (“magnetic Resonance Imaging” or “functional magnetic resonance” or “resting state functional magnetic resonance imaging” or “resting-state fMRI”). Chinese translations of these search terms will be used for the Chinese databases. The search strategy for the PubMed database is shown in Table 1. The search strategy for other online databases will be adjusted according to their requirements.

**Table 1:** The search strategy for PubMed database.

Number	Search terms
#1	Randomized controlled trial. [pt]
#2	Randomised. [ti,ab]
#3	Placebo. [ti,ab]
#4	Randomly. [ti,ab]
#5	Sham. [ti,ab]
#6	Trial. [ti,ab]
#7	Clinical trials as topic. [MeSH]
#8	#1 OR #2 OR #3 OR #4 OR #5 OR #6 OR #7
#9	Human. [MeSH]
#10	#8 AND #9
#12	Insomnia. [MeSH]
#13	Dyssomnias. [MeSH]
#14	Sleep disorders. [MeSH]
#15	Sleep loss. [ti,ab]
#16	Sleep insufficient. [ti,ab]
#17	Sleep deprivation. [ti,ab]
#18	#12 OR #13 OR #14 OR #15 OR #16 OR #17
#19	Depression.
#20	Anxiety.
#21	Emotional disorders.
#22	#19 OR #20 OR #21
#23	#18 AND #22
#24	Acupuncture therapy. [MeSH]
#25	Acupuncture. [ti,ab]
#26	Acupoints. [ti,ab]
#27	Body acupuncture. [ti,ab]
#28	Scalp acupuncture. [ti,ab]
#29	Manual acupuncture. [ti,ab]
#30	Auricular acupuncture. [ti,ab]
#31	Ear acupuncture. [ti,ab]
#32	#24 OR #25 OR #26 OR #27 OR #28 OR #29 OR #30 OR #31
#33	Magnetic Resonance Imaging. [MeSH]
#34	Functional magnetic resonance. [ti,ab]
#35	Resting state functional magnetic resonance imaging. [ti,ab]
#36	Resting-state fMRI. [ti,ab]
#37	MRI. [ti,ab] OR fMRI. [ti,ab] OR BOLD-MRI. [ti,ab] OR rs-fMRI. [ti,ab]
#38	#33 OR #35 OR #36 OR #37
#39	#11 AND #23 AND #32 AND #38

#### 2.2.2 Searching other resources

The reference lists of all included studies or relevant reports of clinical trials or reviews will be screened for additional relevant articles. The WHO International Clinical Trials Registry Platform, Chinese Clinical Trial Registry and ClinicalTrials. Gov, will be searched for ongoing or have finished trials with unpublished data.

### 2.3 Data collection and analysis

#### 2.3.1 Selection of studies

**Figure 1:**
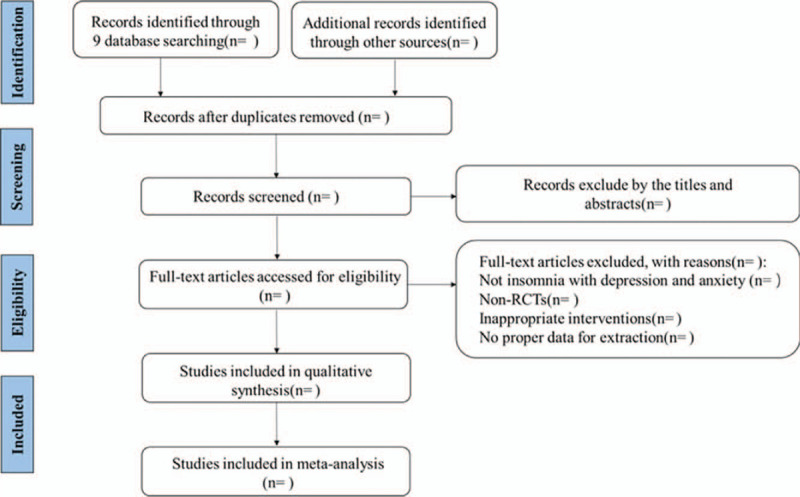
PRISMA flow diagram of the study process. PRISMA = Preferred Reporting Items for Systematic Reviews and Meta-Analyses.

Clinical studies will be identified and reviewed by 2 independent reviewers (LY and ML). In order to ensure that the reviewers have a good understanding of the background and purpose of the review, they will be trained in advance. Use NoteExpress 3.2.0 software (Available at: http://www.inoteexpress.com/aegean/) to independently manage the search results from above-mentioned databases. Firstly, remove the duplicate literature. Secondly, after reviewing the title and abstract, excluding the literature that does not comply with the standard. Finally, further review the full text and rule out the literature that is not line with the standard. Any disagreements related to study selection results will be discussed after cross-checking and arbitrated by the third reviewer (HZ). The flow diagram of all study selection procedure is shown in Figure 1.

#### 2.3.2 Data extraction and management

Two reviewers (JC and YL) will independently screen the study and extracted data, and if there were differences of opinion, the third reviewer (HH) intervened to discuss. According to the designed data extraction table, the basic information of the study including:

1. identification information (article name, publication year, first author);
2. general information (country, study type, sample size, and study design);
3. participants (age, sex, and scale and fMRI results before treatment);
4. interventions (type of acupuncture, acupuncture points selection, and treatment frequency/session/duration);
5. comparator (if there is any, details of the treatment including therapeutic method, dosage, frequency, and course); and
6. outcomes (time, scale and fMRI results points for each measurement, including the Pittsburgh Sleep Quality Index, Insomnia Severity Index , Hamilton Depression Scale , Hamilton Anxiety Scale, ReHo and ALFF, as well as follow-up duration, and adverse events).

#### 2.3.3 Addressing missing data

For study with missing data or clarification for unclear information, we will try to contact corresponding authors for further information. Otherwise, we will analyze the existing information and conduct sensitivity analysis to address the potential impact of missing data.

#### 2.3.4 Assessment of risk of bias in included studies

According to the bias risk assessment tool of Cochrane system reviewer's Handbook V.5.1.0, the bias risk of included literatures will be evaluated by 2 reviewers (LY and ML) independently evaluated each of the following items as “low risk,” “high risk,” or “unclear risk”:

1. selection bias (random sequence generation and allocation concealment),
2. performance bias (blinding of participants and personnel),
3. detection bias (blinding of outcome assessment),
4. attrition bias (incomplete outcome data),
5. reporting bias (selective reporting),
6. other bias (such as presample size estimation, early stop of trial).

#### 2.3.5 Quality of evidence assessment

According to Grading of Recommendations Assessment, Development, and Evaluation (GRADE) method,^[21]^ 2 reviewers (JC and YL) will use the GRADE rating standards to independently assess the quality of evidence for each outcome as 4 levels: “high,” “moderate,” “low,” or “very low” quality. The quality of evidence quality will be assessed according to the risk of bias, inconsistency, indirectness, imprecision, and publication bias.

#### 2.3.6 Data synthesis and analysis

RevMan software (Version 5.3) (Available at: https://community.cochrane.org/help/tools-and-software/revman-5) and stata13.1 (Available at: https://www.stata.com/new-in-stata/meta-analysis/) will be used for meta-analysis. The count data were represented by relative risk and continuous variables by standardized mean difference. 95% confidence interval (95% CI) was calculated for both. The heterogeneity of the included studies was judged by *I*^2^ value, with *P* < .05 as the statistical difference. *I*^2^ values of 0% to 24.9%, 25% to 49.9%, 50% to 74.9%, and 75% to 100% respectively indicate zero, low, medium, and high heterogeneity. When *P* ≥ .10 and *I*^2^ < 50%, fixed effect model was used to analyze the heterogeneity. Otherwise, metaregression analysis was used to analyze the source of heterogeneity. If clinical heterogeneity could not be excluded, random effect model (mantel-Haenszel) was selected. The bias of the study was analyzed by Egger test and funnel plot (*P* < .05).

#### 2.3.7 Subgroup analysis

Subgroup analysis will be conducted to evaluate the specific influence of intervention type, acupoints selecting, acupuncture manipulatio, treatment duration on pooled results. If the data is insufficient, qualitative synthesis will be conducted instead of quantitative synthesis.

#### 2.3.8 Patients and public involvement

There was no patient or public participation in the formulation of the study protocol. Patients will not participate in data collection and analysis of systematic review and meta-analysis.

#### 2.3.9 Ethics and dissemination

Since this meta-analysis will use data from published studies to extract data without involving individuals or privacy, there is no need for ethics approval or patient consent. The results of this meta-analysis will be published in peer-reviewed scientific journals.

## 3 Discussion

Long term insomnia or low sleep quality will have harmful effects on the spirit and cognition of patients,^[22]^ resulting in decreased alertness and increased the occurrence of accidents, errors, and injuries.^[23,24]^ Healthcare support personnel and healthcare practitioners represent 2 of the 5 occupational groups with the highest prevalence of short sleep duration.^[25]^ Nearly 30% of resident physicians and 20% of hospital-based nurses suffer from depression, a rate 2 to 3-fold higher than the general public.^[26]^ A number of studies have confirmed the effectiveness of acupuncture in treating insomnia comorbid with emotional disorders, and acupuncture has the characteristics of simple, convenient, and nontoxic side effects. Therefore, acupuncture therapy is more and more widely used to improve the symptoms of patients with insomnia and the impact on life and work.

With the rapid development of imaging technology, such as Electroencephalogram, Transcranial Magnetic Stimulation, Computed Tomography, Magnetoencephalography, Positron Emission Tomography, and now the most popular Functional Magnetic Resonance Imaging. The development of these technologies has made a greater breakthrough in the positioning and integration of brain function. Rs-fMRI has been widely used in the mechanism of acupuncture treatment of diseases. Research shows that acupuncture can regulate the emotion of insomnia patients, the functional activities of cognitive brain area and the functional connection of functional brain network.^[27]^ In rs-fMRI, the ALFF method can accurately reflect the spontaneous activity of neurons in resting state from the point of view of energy,^[28]^ the increase of ALFF indicates the increase of spontaneous activity in this brain region, on the contrary, it indicates that the spontaneous activity is decreased. ReHo is used to measure the local consistency of spontaneous blood oxygen level dependence in specific brain regions in the resting state, and can stably reflect the time synchronization of local brain neuronal activity.^[29]^ These 2 methods can be used to explore the characteristics and rules of spontaneous brain activity changes in patients, in order to reveal the relevant mechanism.

In recent years, there have been studies on the application of rs-fMRI to explore the mechanism of acupuncture in the treatment of insomnia comorbid with depression and anxiety, but no related systematic review and meta-analysis have been published. This study will include and integrate the latest and most comprehensive literature in this field to explore the effects of acupuncture on brain activity in patients with insomnia comorbid with emotional disorders. However, this study has some limitations. First of all, the relevant research literature is insufficient and may not be able to carry out further correlation analysis, such as subgroup analysis and sensitivity analysis. In addition, there may be heterogeneity between studies due to the use of different evaluation criteria and acupuncture methods. In summary, the purpose of this systematic review and meta-analysis is to provide evidence-based medicine basis for elucidating the central nervous mechanism of acupuncture in the treatment of insomnia comorbid with emotional disorders.

## Author contributions

**Formal analysis:** Lin Yao, Mengyuan Li.

**Investigation:** Lin Yao, Mengyuan Li, Jiazhen Cao, Yanze Liu.

**Methodology:** Lin Yao, Mengyuan Li, Jiazhen Cao, Yanze Liu.

**Project administration:** Hongfeng Wang.

**Resources:** Hongfeng Wang.

**Supervision:** Haizhu Zheng.

**Validation:** Haizhu Zheng.

**Writing – original draft:** Lin Yao.

**Writing – review & editing:** Haipeng Huang, Hongfeng Wang.
